# Review: Examining the Natural Role of Amphibian Antimicrobial Peptide Magainin

**DOI:** 10.3390/molecules25225436

**Published:** 2020-11-20

**Authors:** Katelyn A. M. McMillan, Melanie R. Power Coombs

**Affiliations:** 1 Biology Department, Acadia University, Wolfville, NS B4P 2R6, Canada; kamcmi99@gmail.com; 2 Department of Pathology, Dalhousie University, Halifax, NS B3H 4R2, Canada

**Keywords:** antimicrobial peptide, magainin, African clawed frog, North American green frog

## Abstract

Host defense peptides (HDPs) are a group of antimicrobial peptides (AMPs) that are crucial components of the innate immune system of many different organisms. These small peptides actively kill microbes and prevent infection. Despite the presence of AMPs in the amphibian immune system, populations of these organisms are in decline globally. Magainin is an AMP derived from the African clawed frog (*Xenopus laevis*) and has displayed potent antimicrobial effects against a wide variety of microbes. Included in this group of microbes are known pathogens of the African clawed frog and other amphibian species. Arguably, the most deleterious amphibious pathogen is *Batrachochytrium dendrobatidis*, a chytrid fungus. Investigating the mechanism of action of magainin can help understand how to effectively fight off infection. By understanding amphibian AMPs’ role in the frog, a potential conservation strategy can be developed for other species of amphibians that are susceptible to infections, such as the North American green frog (*Rana clamitans*). Considering that population declines of these organisms are occurring globally, this effort is crucial to protect not only these organisms but the ecosystems they inhabit as well.

## 1. Introduction 

Antimicrobial peptides (AMPs) are a widely studied group of molecules that are known to actively inhibit the growth of various cells types. These peptides are naturally derived from many different organisms and many play a role in the host’s innate immune system. For that reason, some AMPs are also referred to as host defense peptides (HDPs) [1]. This review will provide a look into the nature of magainins, a group of AMPs derived from the African clawed frog (*Xenopus laevis)*. This group of peptides includes magainin I, magainin II, caerulein-precursor fragment (CPF), and peptide glycine-leucine-amide (PGLa) [2,3]. A specific focus will be placed on magainin I and magainin II. These peptides’ mechanisms of action and biochemical properties are described in detail. An overall view of the amphibian immune system with an emphasis on the role of AMPs will also be given. A few major frog pathogens are described, along with signs and causes of infection. 

Magainin is an α-helical peptide that is 23 amino acids in length [4]. This peptide was isolated from the dermis of the skin. The presence of magainin in the skin suggests that this peptide plays a significant role in the prevention of infection by invading pathogens because the skin is usually the first line of defense against a pathogen. This peptide has been found to attack cell membranes using the toroidal pore model, in addition to the carpet model in some cases. As a result of the presence of AMPs, the innate immune response is quite effective at preventing infection. Current populations of amphibians are adversely affected by pathogens including *Batrachochytrium dendrobatidis* [5]. Understanding the specific mode of defense against this pathogen and others is important to eventually resolving the decline in these amphibian populations. Drops in amphibian populations might adversely affect other species populations in the same ecosystem, leading to deleterious effects to other organisms. Considering this, studies into the innate immune system of the African clawed frog is warranted. Conservational efforts into other species such as North American frogs will benefit from more knowledge about the amphibian immune system. Considering the African clawed frog is known to be a carrier of some fungal pathogens and actively prevents infection in most cases, understanding how disease is prevented in this species can lead to efforts protecting more vulnerable species of amphibians native to North America [6].

In addition to the conservational reasons for the study of magainin, there is also a clinical relevance. This AMP has shown to be effective against bacterial species that are pathogenic to humans as well as those pathogenic to amphibians [7]. Studies looking at the effects of magainin against pathogens give us insight into their potential use beyond the conservation of frog populations. Studying these AMPs will heighten our understanding of deleterious pathogen defense as these AMPs are significant in the livelihood of organisms. By examining the current literature on the specific mechanism of action of this peptide and its natural role in the respective hosts we can have a better idea on steps to take in protecting these populations from disease.

## 2. Results

### 2.1. Amphibian Immunity

Amphibians are continuously in contact with terrestrial and aquatic environments and are thus exposed to a wide variety of microbes and pathogens. It is therefore paramount that amphibians have an efficacious immune system to help prevent disease. Accounts on the innate and adaptive immune components of amphibians demonstrate just how advanced the amphibian immune response is against specific pathogens [8]. Arguably, the most important component of the immune system in these organisms is the skin, acting as a barrier to invasion by pathogens. Organisms in the order Anura, including frogs, have evolved various skin adaptations to effectively prevent infection and predation by microbes and predators, respectively, in aqueous and terrestrial habitats [8]. Among these adaptations are mucous and granular glands [8]. These glands secrete potent AMPs, a component of the innate immune system that helps to prevent infection [2,8,9]. In fact, the most extensively studied component of the amphibian innate immune system is arguably AMPs. Many of these peptides are located in amphibian skin, where first contact is likely made with the pathogen [10]. The main mechanism by which AMPs attack microbes appears to be the disruption of the membrane structure. Some studies suggest that AMPs are more effective against pathogens when used synergistically with other AMPs [11]. For example, the AMPs magainin II and peptide glycine-leucine-amide (PGLa) from African clawed frogs prevent the growth of pathogenic fungal species when used in conjunction [3]. While there are many studies on the innate immune system in amphibians, studies on B and T cells of the adaptive immune system are lacking. That being said, one study isolated dendritic epidermal T cells (DETCs) from the African clawed frog in addition to dendritic cells (DCs) and Langerhans cells (LCs) [12]. This shows that amphibians such as frogs are able to present antigens, thereby activating the adaptive immune system. Further study into the amphibian immune system will help to better understand the immune response to amphibian pathogens.

### 2.2. Amphibian Pathogens

Global populations of amphibians have been declining since the 1960s, which is hypothesized to be the result of habitat destruction and the emergence of new diseases [13]. This has spurred research into the pathogens that are partly responsible for this decline in numbers. Significant amphibian pathogens include species of fungi, bacteria, and viruses. The most notable of these pathogens are *Batrachochytrium dendrobatidis*, *Basidiobolus ranarum*, *Aeromonas hydrophila*, and Ranavirus [3,13]. 

*Batrachochytrium dendrobatidis* (*Bd*) is a pathogenic fungus in amphibians that is likely the most significant cause of global declines of amphibian populations [5]. This pathogen is hypothesized to originate from Asia and was spread internationally by the trade of amphibians such as the African clawed frog [6,14]. This fungus causes chytridiomycosis, a lethal skin disease that disrupts the osmotic balance of amphibians [15]. Infections arise from waterborne zoospores that bind to skin and burrow into the epidermis, causing symptoms such as lethargy, cutaneous erythema, decreased respiration rate, and irregular skin sloughing [15,16]. Studies have shown that this fungus causes the disproportionate loss of electrolytes to water, suggesting that osmotic balance is disrupted at the skin, a significant site of osmotic regulation in amphibians [15,17]. This osmotic imbalance leads to cardiac arrest, which is likely the cause of death in populations of amphibians affected by this disease [17], since the rapid loss of electrolytes demonstrated in late stages of the disease would be sufficient to cause the mortality seen in amphibian populations [15]. Attempts have been made to determine possible treatments for the disease. One such example is the use of electrolyte supplements to treat the osmotic imbalance in infected frogs [17]. Treated frogs showed some recovery, but ultimately died from the disease [17]. This suggests that a treatment for chytridiomycosis would require a way to reverse damage to the skin, in addition to preventing the initial infection. 

Knowledge of the specific immune response to *Bd* in amphibians is somewhat lacking. However, some studies have shown that innate and adaptive immune responses are effective at developing resistance to *Bd* infections [2]. African clawed frogs have a consistent *Bd* prevalence of about 3%, suggesting that this species has an effective immune response to this fungus [6]. Due to this resistance to infection, these frogs have proven to be a suitable species to study the amphibian immune response to *Bd*. AMPs from this species have been tested against *Bd* and show significant inhibitory effects against fungal growth [2,11]. This suggests that AMPs play an important role in the immune response against *Bd*. The colonization of *Bd* zoospores on frog skin is strongly inhibited by peptides derived from the African clawed frog [2]. In addition to this, the species is also able to develop specific immune responses to *Bd* by producing antibodies to the fungus [2]. These immunoglobulins have also been detected in skin mucus of these frogs, which suggests both innate and adaptive immunity is utilized in this species’ resistance to *Bd* [2]. Further research into the specific mechanism of action of *Bd* is still required. 

*Basidiobolus ranarum* and *Aeromonas hydrophila* are other amphibian pathogens that may, with comorbidities, lead to a significant decline in amphibian populations. *Basidiobolus ranarum* is a fungus that still causes disease in declining populations of amphibians but is not as widely studied as *Bd* [18]. Symptoms of infection by *B. ranarum* are similar to *Bd* infection, i.e., sloughing of epidermis, erosions, and ulcers [18]. Amphibian AMPs have been found to inhibit colonization by this fungus [3]. This study found that African clawed frog magainins are able to inhibit the growth of *B. ranarum* [3]. Research into the specific immune response to *B. ranarum* is very limited; therefore, more research is required to investigate the mechanism of action of amphibious AMPs against this fungus. Similarly, the immune response to *A. hydrophila* has not been extensively studied. *Aeromonas hydrophila* is an opportunistic bacterium that is found in various species of frogs [19]. This bacterium has been isolated from both healthy and diseased frogs and is known to cause disease in African clawed frog [19]. This bacterium has been shown to be completely resistant to various amphibious AMPs including magainin I, magainin II, PGLa, and CPF [3]. In addition to fungal and bacterial pathogens, amphibians are also susceptible to viral pathogens from the Ranavirus genus in the family Iridioviridae [20]. Frog virus 3 (FV3) is a particularly pathogenic virus from this genus that infects frogs [20]. FV3 is a dsDNA icosahedral virus with a particle diameter of about 130 nm [21]. Adult amphibians that die from FV3 tend to exhibit symptoms such as edema and hemorrhages, which are indicators of kidney damage [22,23]. These fungal and bacterial species are both deleterious for amphibians, despite the bacterial species being primarily opportunistic.

Some amphibian species are able to withstand the pathogenic effects and act as reservoir hosts. The African clawed frog has been shown to act as a reservoir host for Ranavirus species and the fungal species *Bd* [6,24]. By studying components of this organism’s immune system, we can better understand how to protect other amphibian species from infection. Studying how their AMPs can prevent infection would be valuable in understanding how to protect more vulnerable frog species in North America. By understanding the biochemical mechanisms of the peptide, that knowledge can be applied toward developing effective conservational efforts for local species of frogs such as the North American green frog (*Rana clamitans*). 

The North American green frog is an amphibian species commonly found on the eastern coast of North America and is known to be susceptible to the amphibian pathogens Ranavirus and *Bd* [25,26]. Chytridiomycosis is widespread in frog species, with preserved green frog specimens showing a relatively high prevalence of the disease [27]. This prevalence was not enough to indicate widespread death but supported the idea that populations of green frogs are still susceptible to the disease. The presence of other AMPs in the skin of the green frog might explain why detrimental population death does not occur. AMPs from the peptide families ranatuerin-1, ranatuerin-2, ranalexin, and temporin families that were isolated from the skin of green frogs exhibit antimicrobial effects against *Escherichia coli*, *Staphylococcus aureus*, and *Candida albicans* [28]. Amino acid sequences of these peptides are somewhat conserved across other species of frogs from the genus *Rana* [28]. Research on the role of these peptides in the green frog’s defense against infection is lacking and warrants further investigation into their susceptibility to disease. By investigating the green frog’s susceptibility to disease and magainin’s suitability as a preventative treatment for pathogens, these populations of amphibians in the east coast of North America can be protected. 

### 2.3. AMPs’ Mechanism of Action

**Figure 1:**
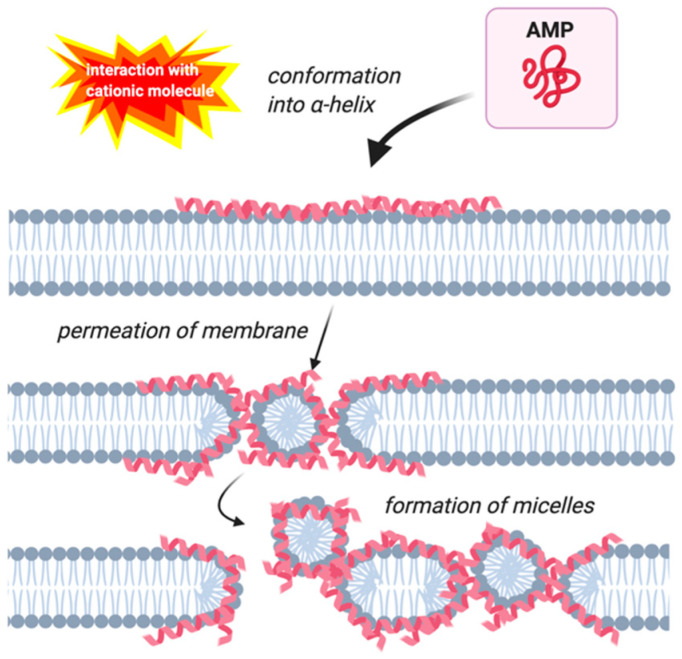
Membrane permeation by the carpet model. Antimicrobial peptides (AMPs) are indicated by pink helices. Image created with BioRender.com.

**Figure 2:**
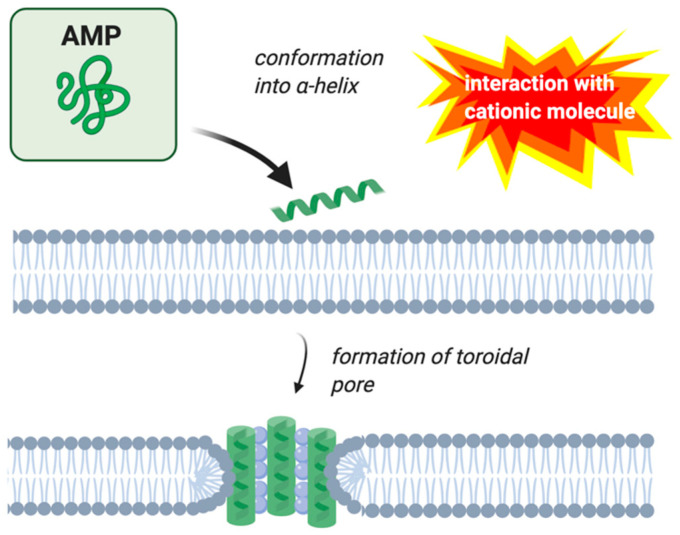
Membrane permeation by the toroidal pore model. AMPs are indicated by green helices. Image created with BioRender.com.

AMPs’ mechanism of action involves disrupting the cell membrane, interfering with cell metabolism, and targeting different cytoplasmic components [29,30]. There are four modes of action that have been described to perform these functions: the barrel-stave, carpet, toroidal-pore, and detergent models [29]. Early investigations on magainin concluded that this peptide acts according to the carpet model (Figure 1), but subsequent research showed that magainin more often acts according to the toroidal pore model (Figure 2) [31,32].

The carpet model involves the AMPs accumulating on the cell surface through electrostatic interactions with hydrophilic amino acids and the phospholipid heads [33]. The peptides only interact with the hydrophilic surface of the membrane, not the hydrophobic interior [30]. Membrane permeabilization only occurs by this model when there is a large concentration of peptides localized around the membrane [34]. Assuming these conditions, the hydrophilic portion of the helical peptides are in contact with the phospholipid head groups during the complete membrane permeabilization process. At no point do the lytic peptides insert into the lipid bilayer [34]. The process begins with the accumulation of the peptides on the surface of the cell, covering the membrane like a carpet [34]. At high peptide to lipid ratios, the peptides begin to form holes in the membrane, which allow the helical AMPs contact to the internal side of the lipid bilayer [30]. Eventually, the accumulated AMPs form micelles between membrane pores (Figure 1) [34].

The toroidal-pore model of permeation involves the phospholipids continuously bending from the outer layer to the inner layer to form pores in the shape of a toroid [30]. The pores are lined not only by the AMPs but also by the head groups of the lipid bilayer [33]. As shown in Figure 2, the pores are well organized with the helical peptides perpendicular to the bilayer on the inside of the pore. Like other pores formed by AMPs, the inner surface of the pore is lined by hydrophilic components, which in this case are both the lipid head groups and polar residues of the peptide. The hydrophobic residues of the peptide face away from the interior of the pore. A characteristic of toroidal pores is the “flip-flop” of lipids across the cell membranes [32,35]. 

### 2.4. Magainin

**Figure 3:**
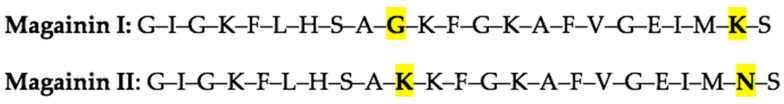
Amino acid sequence of magainin I and magainin II [36]. Two locations of mismatched amino acids on the peptide sequence are highlighted in yellow.

**Figure 4:**
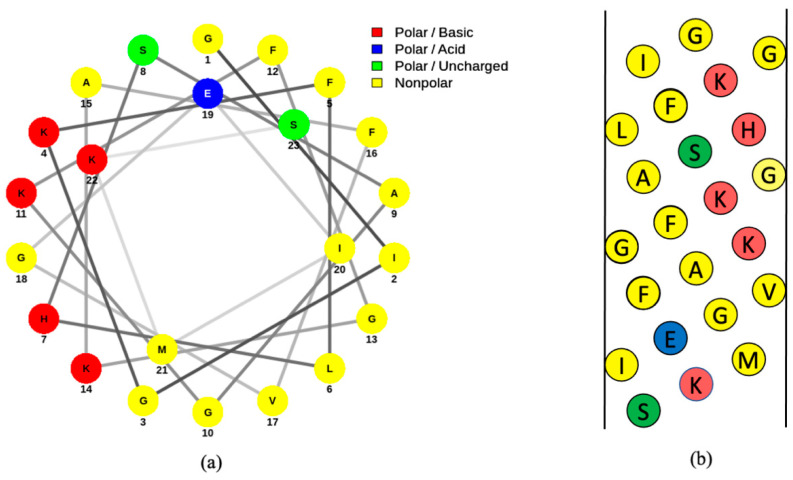
Helical wheel (a) and net diagrams (b) of magainin I [42]. Amino acids are indicated by their single-letter abbreviations and are categorized according to the following colors: polar basic (red), polar acidic (blue), polar uncharged (green), nonpolar (yellow). Both the wheel and net diagrams demonstrate the division of hydrophobic and hydrophilic components of the peptide. The helical wheel diagram was generated using online software called *NetWheels* [43] and the net diagram was made using Microsoft PowerPoint.

Magainins are some of the more extensively-studied amphibian AMPs and were first isolated from the skin of the African clawed frog [36]. This species is considered to be a carrier of *B. dendrobatidis* and, despite being native to Africa, has been found worldwide due to trading [6,37,38]. This frog species is considered to be non-selective in its prey, considering that a wide range of invertebrates have been found in stomach contents of the frog [39]. Magainins are located deep in the dermal tissue [36]. Magainin I and magainin II have near identical amino acid sequences, which are shown in Figure 3 [36]. These magainins are 23 amino acids in length and conform to an alpha helix upon interacting with a membrane [4]. This conformation is likely extended from amino acid residues 2 to 20 [4]. Like many antimicrobial peptides, magainin has an amphipathic structure with hydrophobic residues on one side of the helix and polar residues on the opposite side (Figure 4). These amphipathic properties of the peptide allow for the accommodation of both the hydrophobic and hydrophilic interactions once the peptide is in contact with the phospholipid bilayer [40]. Magainin I and magainin II are considered the most active peptides involved with the immune response in African clawed frogs and have similar antimicrobial properties against various species of Gram-positive and Gram-negative bacteria [36,41]. Magainin I and magainin II do not have significant homology with any other known peptides [36]. 

In addition to antimicrobial action against bacteria, magainins are effective at inhibiting the growth of some species of fungi; acting with an MIC of approximately 162 µM against *Bd* [2]. This broad-spectrum activity allows for the frog to actively fight infections upon first exposure. These frogs can also fully recover from lacerations while exposed to a microbe-rich environment, further suggesting that magainins actively fight infectious pathogens [44]. 

Magainin usually attacks membranes with the toroidal pore model [32,45,46,47]. This peptide has been hypothesized and observed to form a supramolecular pore of peptides and lipids, which is consistent with the pore structure of this model [45]. As described previously, the toroidal pore model of membrane permeabilization involves the pore being lined with both the α-helical peptides and the lipid head groups with the shape of the pore itself resembling a toroid [33]. Magainin’s ability to form heterodimers is thought to be involved with this pore formation [48]. The size of pores formed by magainin in bacterial membranes has been determined to be approximately 2-3 nm in diameter [32]. This ability for pore formation relies on a few conditions in the cell membrane and may result in toroidal-pore formation and even the carpet mechanism [45]. Assuming the presence of these conditions, magainin is a very effective AMP.

Most studies on magainin’s mechanism of action look at the effect on bacterial cells, with some studying the effect on mammalian cells. Considering that some of the most significant pathogens of amphibians are fungi, research on the peptide’s specific interaction with these fungal pathogens is lacking. That being said, one study determined that magainin II strongly inhibits the growth of *B. dendrobatidis*, the cause of chytridiomycosis [2]. In addition to amphibian fungal pathogens, magainin is also an effective antimicrobial agent against *Candida albicans*, a human fungal pathogen that infects the female reproductive tract [7]. Magainin also exhibits sperm immobilizing activity in a variety of mammals, including humans, suggesting the peptide’s potential use as a contraceptive, as well as treating infections [49,50]. In addition to the potential clinical applications of this peptide, future areas of study should include detailed investigations on the specific interaction between magainins and fungal pathogens.

Knowing that magainin usually operates under the toroidal model, further investigations of magainin’s mechanism of action show that magainin interacts with membranes differently depending on their origin and lipid composition. Indeed, this characteristic is present in other AMPs as well. These differences are due in part to the presence of different phospholipids, the protein to lipid ratio, and the cell type. There are many different species of phospholipids, including phosphatidylcholine (PC), phosphatidylglycerol (PG), phosphatidylserine (PS), and phosphatidylethanolamine (PE) to name a few. Depending on the charge of these phospholipids, AMPs may or may not be effective. Since most amphipathic AMPs have an overall positive charge, cell membranes with an abundance of anionic phospholipids such as PS and PG would likely be more susceptible to the AMP. One study determined that the curvature strain of the lipid bilayer significantly affects how magainin interacts with lipid bilayers [45]. With the incorporation of PE, a zwitterionic phospholipid, the curvature strain of the bilayer became more negative and magainin was unable to form toroidal pores [45]. Instead, the peptide disrupted the bilayer by the carpet model [45]. Magainin II is a somewhat effective inactivator of virions by this mechanism [51]. Additionally, the peptide attacks mammalian cells using this mechanism, suggesting PE might be more abundant in this cell type [32]. The permeabilization of these cell membranes by the peptide does not involve adenosine triphosphate (ATP) and glycosaminoglycans [32]. This suggests that magainin is able to permeate cells more readily, given that the peptide does not rely on the presence of other molecules to form toroidal pores. 

**Figure 5:**
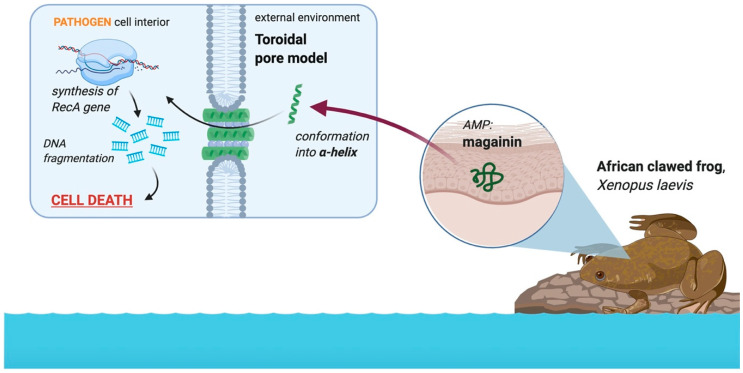
Role of the AMP magainin in the African clawed frog in the defense against amphibian pathogens. The peptide is located in the dermis of the African clawed frog skin and conforms into an alpha helix upon interacting with a negatively charged membrane, resulting in the formation of toroidal pores in most instances. The peptide interferes with internal metabolic processes by activating the RecA gene, which causes DNA fragmentation [41]. This ultimately kills the pathogen. Image created with BioRender.com.

In addition to permeating the bacterial cell membrane using toroidal pores, magainin disrupts internal processes of the cell to initiate cell death [32,41]. Figure 5 illustrates how magainin is able to induce apoptosis-like cell death in bacteria by activating the RecA gene, a gene involved in the regulation of apoptosis [41]. This cell death is initially caused by membrane disruption accompanied with DNA fragmentation, chromatin condensation, and PS externalization [41]. Fluorescence techniques are commonly used in studies on these effects and show that magainin effectively disrupts the membrane integrity of bacterial cells [41]. Despite the lack of studies outlining the specific biochemical pathways involved, there is a significant volume of published research on magainin’s carpet and toroidal mechanism of action that will hopefully lead to more research fully elucidating the peptide’s effect on pathogens. This will help to further understand how the African clawed frog is able to defend against significant diseases such as chytridiomycosis, among others.

## 3. Discussion

The structure and role of AMPs have been investigated for decades, with these peptides being found in many different animals and plants. Magainin, an AMP found in the African clawed frog, was first described in 1987 as being 23 amino acids in length and isolated from the dermis of this frog species [36]. Continual research has been conducted on magainins in the decades after their initial discovery. As a result, the peptides’ mechanism of action against cell membranes has been observed to be mainly the toroidal pore model (Figure 2) and the carpet model (Figure 1) in some situations [32,51]. This review outlined the current research into magainin’s mechanism of action against the cell membrane and the internal processes of the cell. Amphibian pathogens were also described in terms of their pathogenesis and clinical symptoms. Only four pathogens were described here, so this review does not inform on all amphibian pathogens. There is a need to better understand the amphibian immune system and the role of amphibian AMPs in immune defense toward amphibian pathogens. There are potentially other pathogens that are more significant than *Bd*. Additionally, pathogens are not the only variable involved in amphibian death and the expression of AMPs with environmental factors needs to be considered to better understand population declines and ultimately to protect these vulnerable populations. 

Due to the prolonged research effort into AMPs, there is a significant body of work on the role magainins play in the African clawed frog, including their specific mechanism of action against *Bd*. A review published in 1999 by Daszak et al. reviewed the findings of the primary research articles that investigated *Bd* after being first described in 1998 [13]. The clinical signs of infection by this pathogen were described in detail, as well the emergence of the pathogen worldwide. Through these detailed and broad accounts of amphibian mortality, this paper clearly demonstrated the need for further research into disease caused by emerging pathogens and how amphibians protect against them. Another paper described the amphibian defenses against *Bd* by investigating the role of host defense peptides and the effect of a depleted immune system on the infection of African clawed frogs with *Bd* [2]. This paper gives valuable insight into the components of the amphibian immune response that fight against fungal pathogens as well as the resistance of the African clawed frog against high levels of *Bd* [2]. By continuing to study how this frog species is able to defend against the pathogen, we can better understand how to apply these immune components to other species, potentially through developing a preventative treatment comprised of amphibian AMPs. Matsuzaki et al. in 1996 proposed the toroidal pore model to explain the mechanism of action of magainin II using large unilamellar vesicles to demonstrate that magainin II forms transmembrane pores lined with the polar side of the helical peptide facing the interior of the pore [31]. Subsequent research into magainin’s mechanism of action further supports this conclusion.

Despite this current volume of research on magainin and other AMPs, more work needs to be done to completely elucidate magainin’s mechanism of action. Methods such as fluorescence spectroscopy, circular dichroism spectroscopy, and macroscopic imaging are among the common experimental methods used to investigate the mechanism of action of this peptide [31,45,46,52]. Despite use of these cutting-edge methods, there are still gaps in the knowledge of magainin’s mechanism of action. Considering the peptide typically acts according to the toroidal pore model, the observations that the peptide conforms to the carpet model in some conditions presents a discrepancy in completely understanding the peptide’s role in membrane attack. More research needs to be done to completely understand the conditions that permit the peptide to attack by either model. Doing so will enable a better understanding of how amphibian peptides may be used as a preventative treatment against varying amphibian pathogens. 

Future directions in this area of research include optimizing the peptide’s function and developing a preventative treatment for amphibian populations that can prevent infection by deleterious pathogens. Amino acid substitutions may be carried out on the magainin peptide to further enhance its antimicrobial action and to make the peptide compatible for treating other species of amphibians. Considering that *Bd* is arguably the most significant pathogenic culprit regarding the fall in amphibian populations, these future studies would focus on optimizing the action of the peptide in regard to this particular pathogen. Additionally, other deleterious pathogens that are resistant to amphibian AMPs should be investigated. Efforts should also focus on investigating how these AMPs interact with the host immune system during an immune response. Determining the role of AMPs in various species of amphibians will help understand how certain species of amphibians can protect against infection better than others. 

## 4. Materials and Methods 

The database Pubmed was primary used to find the studies included in this review. The database Environment Complete was also used to find relevant studies specific to the conservational aspect of amphibian species in North America. The key terms used in these searches were: magainin, *Xenopus laevis*, African clawed frog, antimicrobial peptide, amphibian pathogen, *Batrachochytrium dendrobatidis*, Ranavirus, *Basidiobolus ranarum, Aeromonas hydrophila*, green frog, and *Rana clamitans*. 

Three types of software were used to make the figures included in this publication. *NetWheels* software [43] was used to plot the helical wheel representation of magainin I. Microsoft PowerPoint was used to construct the net diagram of magainin I and the amino acid sequences of magainin I and magainin II. Lastly, Images in Figure 1, Figure 2, and Figure 5 were made in ©BioRender-biorender.com.

## 5. Conclusions

Global populations of amphibians are in decline, partly due to their infection by a variety of pathogens. Studying the role of AMPs in the immune response to these pathogens is paramount in developing a treatment for these populations to prevent further infections. Most notable among amphibian pathogens is *Batrachochytrium dendrobatidis (Bd)*, a chytrid fungus. The African clawed frog contains magainin AMPs, which have been shown to actively display antimicrobial effects against these amphibian pathogens, in addition to human pathogens. This frog species is particularly effective in defending against infection by *Bd* through both the innate and adaptive immune system. The host defense peptide magainin is thought to play a significant role in this prevention of infection. The peptide magainin typically attacks cell membranes of pathogens through the toroidal pore method and the carpet model under certain conditions, in addition to altering the internal metabolism of the pathogens. By continuing research into understanding the role of other amphibian AMPs, factors that impact their expression and their impact on innate immunity, we may be able to implement preventive strategies to prevent further amphibian species population decline.
